# Photoluminescence Investigations of Dy^3+^-Doped Silicate Xerogels and SiO_2_-LaF_3_ Nano-Glass-Ceramic Materials

**DOI:** 10.3390/nano12244500

**Published:** 2022-12-19

**Authors:** Natalia Pawlik, Tomasz Goryczka, Ewa Pietrasik, Joanna Śmiarowska, Wojciech A. Pisarski

**Affiliations:** 1 Institute of Chemistry, University of Silesia, 40-007 Katowice, Poland; 2 Institute of Materials Engineering, University of Silesia, 41-500 Chorzów, Poland

**Keywords:** Dy^3+^ ions, SiO_2_-LaF_3_ nanocrystalline systems, sol-gel technique, visible light emission

## Abstract

In this work, the series of Dy^3+^-doped silicate xerogels were synthesized by sol-gel technique and further processed at 350 °C into SiO_2_-LaF_3_:Dy^3+^ nano-glass-ceramic materials. The X-ray diffraction (XRD) measurements, along with the thermal analysis, indicated that heat-treatment triggered the decomposition of La(TFA)_3_ inside amorphous sol-gel hosts, resulting in the formation of hexagonal LaF_3_ phase with average crystal size at about ~10 nm. Based on the photoluminescence results, it was proven that the intensities of blue (^4^F_9/2_ → ^6^H_15/2_), yellow (^4^F_9/2_ → ^6^H_13/2_), and red (^4^F_9/2_ → ^6^H_11/2_) emissions, as well as the calculated yellow-to-blue (Y/B) ratios, are dependent on the nature of fabricated materials, and from fixed La^3+^:Dy^3+^ molar ratios. For xerogels, the emission was gradually increased, and the τ(^4^F_9/2_) lifetimes were elongated to 42.7 ± 0.3 μs (La^3+^:Dy^3+^ = 0.82:0.18), however, for the sample with the lowest La^3+^:Dy^3+^ molar ratio (0.70:0.30), the concentration quenching was observed. For SiO_2_-LaF_3_:Dy^3+^ nano-glass-ceramics, the concentration quenching effect was more visible than for xerogels and started from the sample with the highest La^3+^:Dy^3+^ molar ratio (0.988:0.012), thus the τ(^4^F_9/2_) lifetimes became shorter from 1731.5 ± 5.7 up to 119.8 ± 0.4 μs. The optical results suggest, along with an interpretation of XRD data, that Dy^3+^ ions were partially entered inside LaF_3_ phase, resulting in the shortening of Dy^3+^-Dy^3+^ inter-ionic distances.

## 1. Introduction

For the past few decades, optical materials doped with luminescent rare earths (RE^3+^) have attracted immense attention because of their plenteous application in photonic devices, like displays, lasers, light-emitting diodes (LEDs), and sensors [1,2,3,4]. Among RE^3+^, the visible luminescence of Dy^3+^ ions inside blue (488 nm, ^4^F_9/2_ → ^6^H_15/2_ transition), and yellow (570 nm, ^4^F_9/2_ → ^6^H_13/2_ hypersensitive transition) regions makes Dy^3+^-doped optical materials promising candidates for utilization as white light emitters. For instance, barium silicate glasses doped with Dy^3+^ are suitable for a white light generation defined by chromaticity coordinates equal to (0.31|0.34), which are lying near the standard point for the while illuminant (0.33|0.33) [5]. Similarly, the chromaticity coordinates for Dy^3+^-doped lithium zinc borosilicate glasses (e.g., (0.318|0.357) or (0.321|0.347)) were also found to be located inside the white light region, and the calculated correlated color temperatures (CCT) are above 5700 K, which indicates that the glasses emit cool white light [6]. Further, it was reported that the lithium aluminum borate glasses co-doped with Gd^3+^/Dy^3+^ ions are able to produce neutral white light (0.363|0.402) with CCT equal to 4556 K [7], but the warm white light was obtained for selected Dy^3+^-doped glass-ceramics containing Na_3_Gd(PO_4_)_2_ phase [8]. Moreover, since the populations of the ^4^I_15/2_ and the ^4^F_9/2_ excited levels of Dy^3+^ ions are governed by the Boltzmann statistics, they are thermally coupled, which makes it possible to apply them in optical thermometry. Factually, Bu et al. [9] found for Dy^3+^-doped glass-ceramics containing LaF_3_ crystal phase that the intensities of emissions located at 480 nm (^4^F_9/2_ → ^6^H_15/2_) and 572 nm (^4^F_9/2_ → ^6^H_13/2_) are decreased, but conversely, the luminescence within the blue light scope at 454 nm (^4^I_15/2_ → ^6^H_15/2_) gradually increased as temperature rose from 275 to 550 K. Similarly, Komar et al. [10] stated that the fluorescence intensity ratio between the ^4^I_15/2_ → ^6^H_15/2_ and the ^4^F_9/2_ → ^6^H_15/2_ emission lines for La_3_Ga_5.5_Ta_0.5_O_14_ crystal could be treated as a temperature-dependent parameter up to 773 K. Further, the materials co-doped with Dy^3+^ and transition metals could also be used in the field of luminescence thermometry. Indeed, Lin et al. [11] identified that the ^2^E_g_ → ^4^A_2g_ transition of Mn^4+^ is very sensitive to temperature fluctuations because of the thermal quenching of the luminescence as a result of strong electron–phonon coupling. Contrary, the authors found that the shielded 4f^9^-4f^9^ emissions of Dy^3+^ ions are relatively negligibly affected by the lattice environment and thus, the ratio between emission intensities of Mn^4+^ and Dy^3+^ ions is dependent on temperature.

The spectroscopy of trivalent Dy^3+^ ions is widely described for various types of glassy hosts fabricated by the conventional melt-quenching technique and the derivative glass-ceramics [12,13,14,15,16], as well as phosphors like Li^+^-doped CaWO_4_:Dy^3+^ [17], BaSrY_4_O_8_:Dy^3+^ [18], or SrLaAlO_4_:Dy^3+^ [19], and complexes [20,21,22]. However, the literature concerning optical properties of Dy^3+^-doped sol-gel materials is far less exhausting. In this field, the studies published by the research group of B. Grobelna focused on Dy^3+^-doped silicate xerogels containing selected types of tungstates, e.g., CaWO_4_ [23], and Ln_2_(WO_4_)_3_ (Ln = La or Gd) [24,25]. The characteristic blue (^4^F_9/2_ → ^6^H_15/2_) and yellow (^4^F_9/2_ → ^6^H_13/2_) luminescence of Dy^3+^ ions were generated via the energy transfer from WO_4_^2-^ using an excitation wavelength from the mid-UV area (λ_ex_ = 240 nm). Thus, the authors stated that fabricated optical materials could be potentially used in solar cells to enhance conversion efficiency. Additionally, the spectroscopy of Dy^3+^ ions in sol-gel materials was described for Dy^3+^/Tb^3+^ co-doped 90SiO_2_-10YF_3_ (mol%) [26] and 95SiO_2_-5LaF_3_ (mol%) [27] nano-glass-ceramics, and according to the energy transfer from Dy^3+^ to Tb^3+^ those systems are considered as promising candidates for solar cells applications. The synthesis of zirconate xerogels and aerogels containing Dy^3+^ ions were presented in work [28], and their characterization provided the thermal and structural analysis; however, the luminescence measurements of Dy^3+^ luminescence were not the aim of the presented studies. Therefore, such a relatively small number of papers devoted to Dy^3+^ ions spectroscopy in sol-gel materials makes investigating those types of optical materials highly meaningful and necessary.

Among various types of optical materials based on fluorides (e.g., CaF_2_, SrF_2_, BaF_2_, YF_3_), LaF_3_ is one of the most frequently and willingly studied host, as evidenced by plentiful works reported in the current literature [29,30,31,32,33,34,35]. LaF_3_ is characterized by exceptionally low phonon energy (~350 cm^−1^) arising from the strong ionicity of La^3+^-F^−^ bond, compared with other RE^3+^-F^−^ or Na^+^-F^−^ [36,37]. LaF_3_ crystallizes as the trigonal/hexagonal phase, but the cubic polymorphic form is also known and reported [38,39]. Moreover, LaF_3_ is also characterized by good transmission within the range between 0.13 and 11 μm [40,41]. The similarity in ionic radii of La^3+^ cation and other RE^3+^ dopants allows for relatively easily substituting them in the parent fluoride crystal lattice to improve the optical properties by suppressing the non-radiative losses of photon energy [42]. Thus, these peculiarities clearly point out the great utility potential of glass-ceramic materials containing LaF_3_ crystals doped with optically active RE^3+^ ions. Moreover, in this regard, it should be noted that the fabrication of oxyfluoride glass-ceramic materials using the sol-gel method allows for overcoming the fundamental drawback of the melt-quenching technique, which is often correlated with the high risk of the evaporation of fluorides (even about 30–40 mol%) [43]. The appropriate chemical reactions (i.e., hydrolysis of the metal/semi-metal alkoxide, further condensation, and polycondensation) during sol-gel synthesis are usually performed at room temperature (or slightly elevated); it is used as an alternative route for the fabrication of the oxyfluoride glass-ceramic materials. As a result, this low temperature approach allows for the fabrication of nano-glass–ceramics with a greater fluoride crystal fraction.

In this study, the series of silicate xerogels doped with Dy^3+^ ions were fabricated using the sol-gel technique, and further processed into SiO_2_-LaF_3_:Dy^3+^ nano-glass-ceramics. The molar ratio of La(CH_3_COO)_3_:Dy(CH_3_COO)_3_ acetates used during the sol-gel synthesis was changed as follows (1 − x):x, where x = 0.012, 0.03, 0.06, 0.12, 0.18, and 0.3. The thermal analysis and XRD technique were used to verify the structural transformation during performed controlled heat-treatment of precursor silicate xerogels. The impact of La^3+^:Dy^3+^ molar ratio, as well as the influence of xerogels’ evolution into nano-glass-ceramics on photoluminescence properties was discussed based on excitation and emission spectra, along with the decay analysis from the ^4^F_9/2_ excited state of Dy^3+^ ions.

## 2. Materials and Methods

The sol-gel preparation method used to synthesize the series of xerogels doped with Dy^3+^ was described with details elsewhere [44]. All reagents were taken from Sigma Aldrich Chemical Co. (St. Louis, MO, USA). The subsequent chemical reactions, which undergo during sol-gel evolution, e.g., hydrolysis, condensation, and polycondensation of precursor (tetraethoxysilane, TEOS), were carried out in a solution of ethyl alcohol (EtOH), deionized water, and acetic acid (AcOH), with molar ratio equals to 1:4:10:0.5. In parallel, the appropriate amounts of La(AcO)_3_ and Dy(AcO)_3_ acetates were dissolved in water and trifluoroacetic acid (TFA), and the resultant mixtures were added dropwise to TEOS-based solutions. The molar ratio of TFA:Ln^3+^ (La^3+^ and Dy^3+^) was set at 5:1, which finally varied as follows: TFA:La^3+^:Dy^3+^ = 5:(1 − x):x, where x = 0.012, 0.03, 0.06, 0.12, 0.18, and 0.3. The as-prepared sols were poured into beakers that were kept sealed until rigid xerogels were formed. The sol-gel evolution from the silicate sols, through wet-gels, up to solid xerogels was performed at 35 °C for the next several weeks, and the following samples were denoted as XG1-XG6. The transformation of xerogels into oxyfluoride nano-glass-ceramics was conducted at 350 °C for 10 h. The fabricated SiO_2_-LaF_3_:Dy^3+^ nano-glass-ceramics were marked in the text as GC1-GC6.

The thermogravimetry and differential scanning calorimetry (TG/DSC) were carried out using a Labsys Evo system with a heating rate of 10 °C/min in argon atmosphere (SETARAM Instrumentation, Caluire, France). The prepared sol-gel materials were characterized by X-ray diffraction (XRD) analysis using an X’Pert Pro diffractometer supplied by PANalytical with CuKα radiation with λ = 1.54056 Å wavelength (Almelo, the Netherlands). The luminescence measurements were performed on a Photon Technology International (PTI) Quanta-Master 40 (QM40) UV/VIS Steady State Spectrofluorometer (Photon Technology International, Birmingham, NJ, USA), supplied with a tunable pulsed optical parametric oscillator (OPO) pumped by the third harmonic of a Nd:YAG laser (Opotek Opolette 355 LD, OPOTEK, Carlsband, CA, USA). The laser system was coupled with a xenon lamp, a double 200 mm monochromator, and a multimode UV/VIS PMT detector. The excitation and emission spectra were recorded with a resolution of 0.5 nm. The luminescence decay curves were recorded by a PTI ASOC-10 (USB-2500) oscilloscope with ±0.1 μs accuracy. All structural and optical measurements were carried out at room temperature.

## 3. Results and Discussion

### 3.1. Analysis of TG/DTG and DSC Results for Dy^3+^-Doped Xerogels

**Figure 1:**
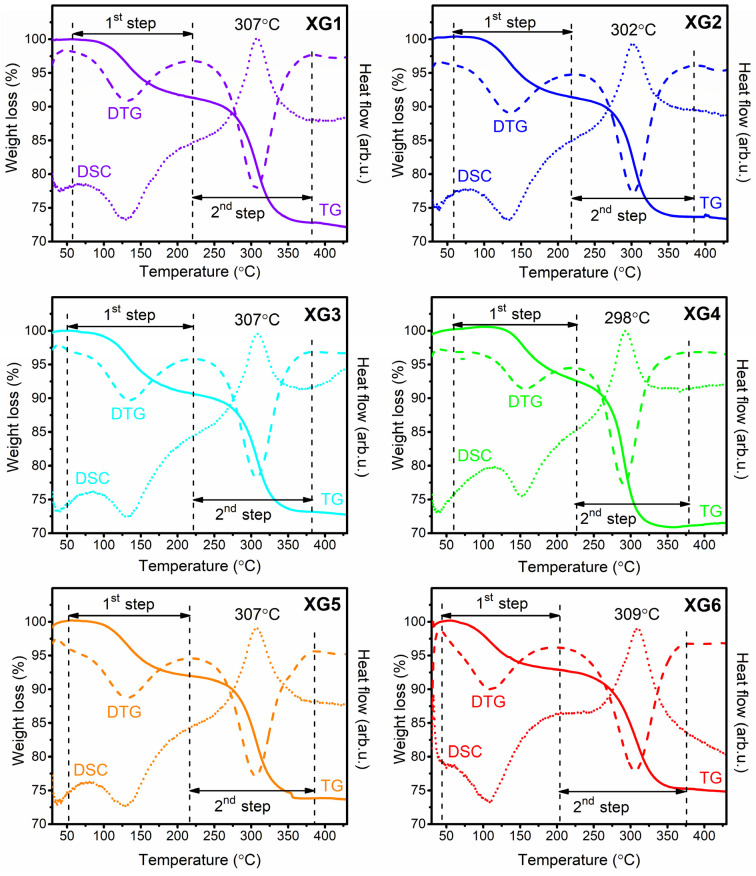
TG, DSC and DTG curves recorded for prepared XG1-XG6 xerogels (presented as solid, dashed, and dotted lines, respectively).

Figure 1 presents the TG/DSC curves recorded for fabricated xerogels in an inert gas atmosphere in a temperature range from 30 to 430 °C. The TG technique involves the measurement of weight losses as a function of temperature; therefore, the TG curves demonstrate the thermal stability of the studies samples. According to the analysis of TG curves (solid lines), there are two distinguishable degradation steps for all xerogels: first, identified within a temperature range from ~55 to ~220 °C, and second, between ~220 and ~380 °C. The indicated degradation steps are marked in Figure 1, and the appropriate temperature ranges for individual xerogels doped with Dy^3+^ ions are collected in Table 1.

The first degradation step, which occurred in lower temperatures (in a range from ~55 to ~220 °C), is correlated with the evaporation of residual organic solvents (ethyl alcohol, acetic acid, unreacted TFA) and water desorption from the porous silicate sol-gel network, and is observed as a gentle degradation. Actually, xerogels are porous solid materials, with pores that are usually filled by liquids. Despite the vibrations characteristic for the silicate sol-gel network (~1200 cm^−1^ and below), there are additional bands identified as the vibrations of OH groups (>3000 cm^−1^), C=O moieties (1650 cm^−1^), and C–H bonds (~1390 cm^−1^, ~1460 cm^−1^), which clearly indicate the presence of water and organic compounds residues in xerogels before any heat-treatment [44]. Indeed, the boiling points of indicated chemical compounds under atmospheric pressure (i.e., 72 °C for TFA, 78 °C for C_2_H_5_OH, 100 °C for H_2_O, and 118 °C for CH_3_COOH). Hence, according to the 1st step of degradation, the evaporation of the compounds mentioned above is expected in a given temperature range. Supplementarily, the derivative thermogravimetry (DTG) expresses the results of TG by providing the first derivative curve as a function of temperature. DTG is a type of thermal analysis in which the rate of xerogels’ mass changes upon heating is plotted against temperature. Therefore, the temperature at which the maximum of the first DTG peak (dashed lines) occurs indicates the temperature at which the evaporation of water and organic compounds undergo the maximum rate. The temperatures from DTG curves for an individual sol-gel sample are identified in a range from 111 °C (XG6) to 157 °C (XG4) and are summarized in Table 1.

According to the processing of nano-glass-ceramic materials, the 2nd step of thermal degradation is essential because it is directly related to the crystallization of the LaF_3_ fluoride phase, preceded by La(TFA)_3_ decomposition. Indeed, this process involved a chemical reaction in which the compounds LaF_3_, (CF_3_CO)_2_O, CO_2_, and CO are obtained. The investigations of the mechanism of this reaction allowed us to conclude that such thermolysis led to cleavage of C–F bonds inside −CF_3_ groups from TFA ligand, and the fluorine anions (F^−^) tend to react with La–O bonds, forming LaF_3_ phase resultantly [45]. From TG analysis (solid lines), the indicated transformation within the structure of prepared sol-gel materials is observable as a significant decrease in the mass. Accordingly, the temperatures at which the maximum of the DTG peaks for an individual sample are identified as approximately 300 °C. The indicated weight losses associated with La(TFA)_3_ thermal decomposition are estimated at 29.76 (XG1), 27.99 (XG2), 27.97 (XG3), 29.54 (XG4), 26.90 (XG5), and 28.02% (XG6). Moreover, for each fabricated xerogel, a strong exothermic DSC peak (dotted line) in this temperature range is recorded with a maximum near 300 °C. Therefore, such a degradation step is according to the release of energy and mass. The location of DSC peaks is consistent with the maxima of DTG peaks, which correspond to temperatures at which the transformation occurs the most rapidly.

The obtained results are consistent with the literature data, which clearly indicate that the thermal decomposition of metal trifluoroacetates and crystallization of appropriate fluoride phases occur at about 300 °C [44,46,47]. Additionally, based on the TG analysis, it should be noted that the prepared sol-gel samples are characterized by good thermal resistance at temperatures close to 350 °C. According to data collected in Table 1, it could be assumed that co-doping with Dy^3+^ ions was not influenced the thermal parameters of the prepared silicate xerogels. Based on collected data from TG (DTG) and DSC measurements, the temperature of a heat-treatment process to fabricate nano-glass-ceramics was assessed at 350 °C.

### 3.2. Structural Characterization of Fabricated Dy^3+^-Doped Sol-Gel Materials

**Figure 2:**
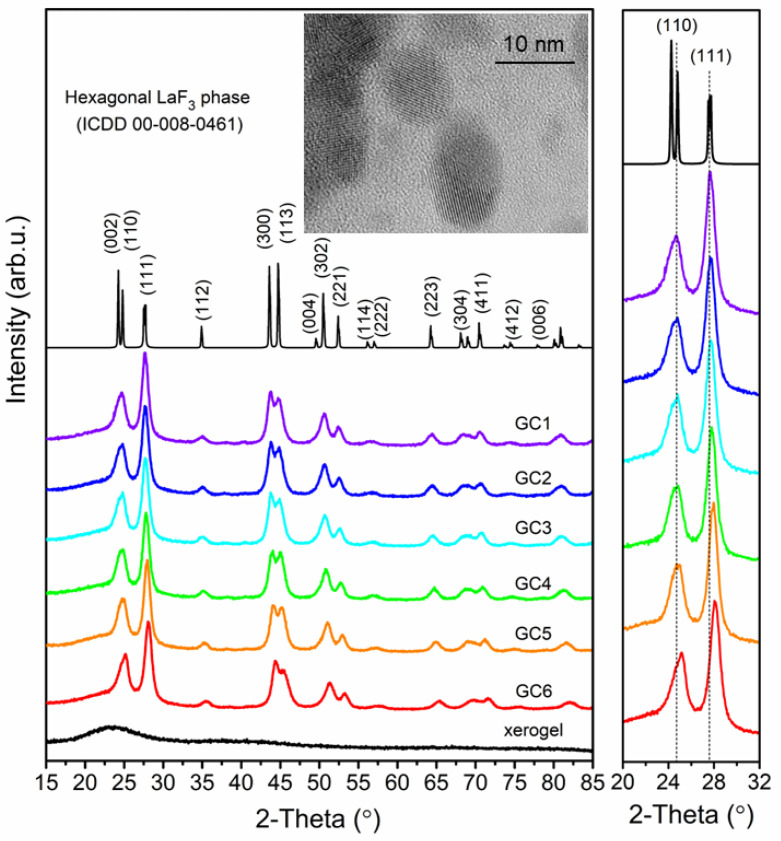
XRD patterns for the series of fabricated sol-gel samples. The region between 20° and 32° was enlarged to show the impact of Dy^3+^ content on diffraction lines shifting. The inset shows HR-TEM image of GC1 sample.

Figure 2 shows the XRD diffractograms of Dy^3+^-doped precursor xerogels and samples obtained during controlled heat-treatment at 350 °C. For xerogels, the XRD patterns showed no sharp diffraction lines but only a broad hump with a maximum located near ~22°, which confirmed their amorphous nature devoid of long-range order [48]. Conversely, the sharp XRD lines are well-visible for heat-treated samples, and the lines are attributed to the hexagonal LaF_3_ phase crystallized in P6_3_cm space group (ICDD card no. 00-008-0461). According to the literature, in the nearest framework around La^3+^ cations, there are nine F^−^ anions with four non-equivalent sites, including 3F1, 3F2, 2F3, and 1F4 [49]. The broadening of the diffraction lines was used to calculate the average diameter (D) of the crystallized LaF_3_ phase using the Scherrer equation [50]:

(1) $$D=\frac{K\lambda }{\beta_{\mathrm{hkl}}\cos\theta }$$

where K is a shape factor (in our calculations it was taken K = 1), λ is a wavelength of X-ray (0.154056 nm, Kα line of Cu), β_hkl_ is a broadening of the (hkl) diffraction peak at half of the maximum intensity, and θ is a Bragg’s angle. The average crystallite size was estimated from 11.9 ± 0.1 (GC6) to 21.3 ± 0.5 nm (GC1). Additionally, the Williamson–Hall theorem was also used to determine the average size of LaF_3_ phase [51]:

(2) $$\beta_{\mathrm{hkl}}\cos\theta =\frac{K\lambda }{D}+4Z\sin\theta$$

in which β_hkl_ is a broadening of the (hkl) diffraction line, θ is a diffraction angle, λ is an X-ray wavelength, D is an average crystal size, and Z is an effective strain. The lattice strain and the crystallite size were deduced from the intercept of βcos θ/λ versus sin θ/λ. The average crystal sizes of LaF_3_ from the Williamson–Hall method are similar for all fabricated SiO_2_-LaF_3_:Dy^3+^ nano-glass-ceramics and were estimated from 8.2 ± 0.1 (GC2) to 10.6 ± 0.1 nm (GC1). As can be seen from the obtained results, there is a noticeable difference in the size of the crystallites obtained by the Scherrer and Williamson–Hall methods. The difference is because the Scherrer method does not consider the share of internal stresses in the half-width of the XRD diffraction line. Contrary, the Williamson–Hall method separates the half-width into parts associated with the average crystallite size and parts related to internal stresses. If there would be no internal stresses in the material, the results of methods are convergent. If there are no internal stresses in the material, the results of the methods are convergent. However, the dysprosium ions caused some internal stress, so the estimated crystallite sizes obtained by these methods are slightly different. In the case of fabricated samples, Dy^3+^ ions, the inset of Figure 2 displays the high-resolution transmission electron microscope (HR-TEM) image of the prepared GC1 sample. Based on it, it was stated that the size of LaF_3_ nanocrystals is consistent with the average crystal size estimated from XRD analysis.

Figure 2 also shows an evident shift of (002), (110), and (111) diffraction lines toward higher angles as the content of Dy^3+^ ions increases in the subsequent samples in the prepared series. The shift in the position of (110) diffraction line (∆θ), compared with pure LaF_3_ phase, is about from 0.01 to 0.40° for GC2 and GC6 nano-glass-ceramics, respectively. These results indicate that the lattice parameters for the cation-exchanged LaF_3_:Dy^3+^ phase are smaller than for the pure fluoride phase without any admixtures of Dy^3+^ ions. So, because Dy^3+^ ions have a slightly smaller ionic radius (r = 1.083 Å) compared with La^3+^ cation (r = 1.216 Å) [52], some lattice distortions and intra-stress occur, as was presented in Table 2. Indeed, a general tendency to a progressive decrease in the cell parameters of fluoride nanocrystals was denoted (from a_0_ = 7.181(8) Å, c_0_ = 7.359(4) Å for GC1 up to a_0_ = 7.077(2) Å, c_0_ = 7.242(9) Å for GC6) in comparison with that of pure and undoped LaF_3_ phase (a_0_ = 7.184 Å, c_0_ = 7.351 Å). So, since the ionic radius of dopant (Dy^3+^) and cation from parent fluoride crystal lattice (La^3+^) are slightly different, the substitution of La^3+^ by Dy^3+^ modifies the inter-ionic distances and induces the perturbation in the lattice parameters. It generates stress inside the nanocrystal lattice, and for LaF_3_:Dy^3+^ system the compressive strain could be observed [53,54]. The lattice strain derived from the Williamson–Hall formula for fabricated sol-gel samples was estimated from 0.11 ± 0.01% to 0.27 ± 0.01%, indicating some lattice distortion. Interestingly, conversely to the above tendency, a very slight increase in c_0_ parameter for GC1 sample (c_0_ = 7.359(4) Å) in comparison with those of the pure LaF_3_ phase (c_0_ = 7.351 Å) was observed. It may be correlated with a peculiar property of crystals in the nanoscale, as was also denoted e.g., for CeO_2_ [55,56], BaF_2_ [57], or Pt nanoparticles [58].

### 3.3. Optical Properties of Dy^3+^-Doped Xerogels

**Figure 3:**
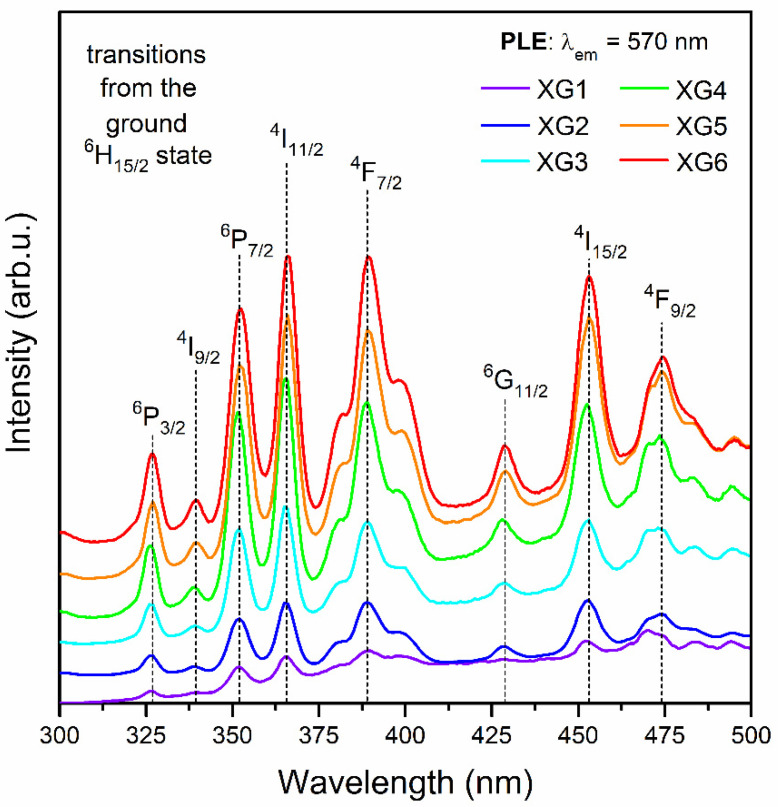
Photoluminescence excitation spectra (PLE) recorded for the series of fabricated xerogels by monitoring the yellow emission at λ_em_ = 570 nm.

**Figure 4:**
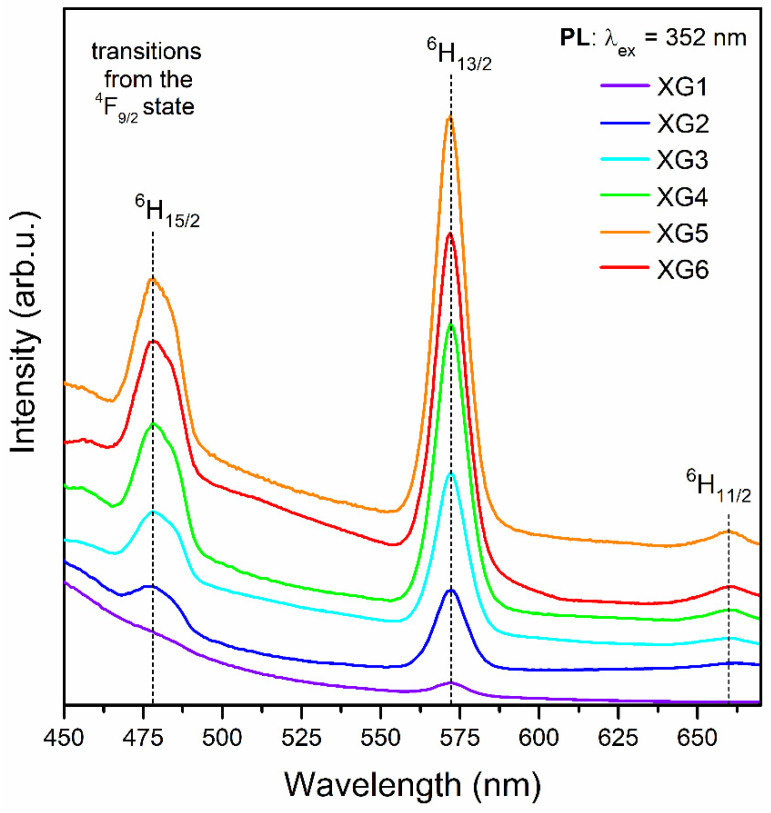
The photoluminescence emission (PL) spectra recorded for the series of prepared xerogels upon near-UV excitation at λ_em_ = 352 nm.

**Figure 5:**
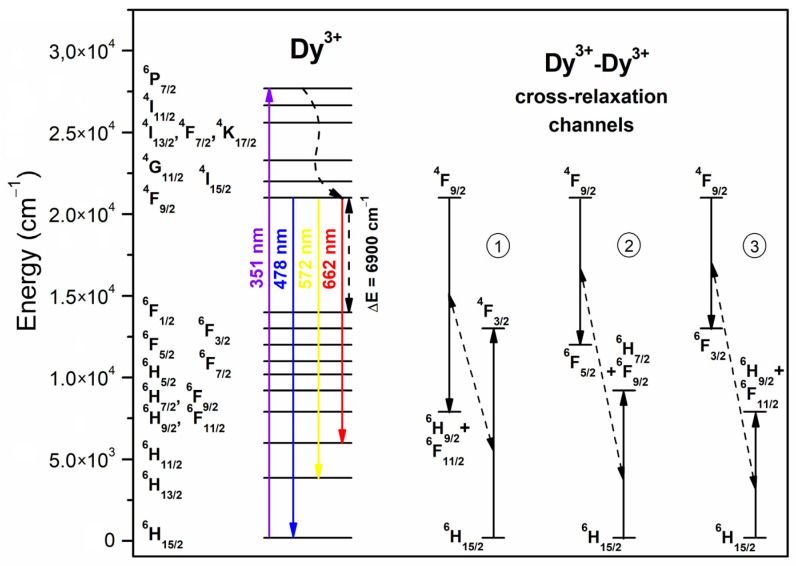
The energy level scheme of Dy^3+^ along with the cross-relaxation (CR) channels.

Figure 3 illustrates the excitation spectra for the series of Dy^3+^-doped xerogels, registered on collecting the yellow emission at λ_em_ = 570 nm. Within the near-UV and VIS ranges, the 4f^9^-4f^9^ intra-configurational transitions originating from the ^6^H_15/2_ ground state of Dy^3+^ ions to the various excited levels were noted, appropriately labeled as the ^6^P_3/2_ (326 nm), ^4^I_9/2_ (340 nm), ^6^P_7/2_ (352 nm), ^4^I_11/2_ (366 nm), ^4^F_7/2_ (388 nm), ^6^G_11/2_ (427 nm), ^4^I_15/2_ (452 nm), as well as ^4^F_9/2_ (474 nm). It could be observed that the intensities of individual excitation bands have grown with decreasing La^3+^:Dy^3+^ molar ratio as the content of Dy^3+^ ions increased. On the other hand, since the intensities of excitation bands for XG5 and XG6 samples are comparable, it could be stated that the energy transfer processes between Dy^3+^ ions started to occur, suggesting the concentration quenching. The emission spectra of Dy^3+^-doped xerogels are presented in Figure 4. The spectra were recorded upon excitation at λ_ex_ = 352 nm and show three luminescence bands at 477, 570, and 655 nm, according to the following transitions: ^4^F_9/2_ → ^6^H_15/2_ (blue), ^4^F_9/2_ → ^6^H_13/2_ (yellow), and ^4^F_9/2_ → ^6^H_11/2_ (red), as was also presented in the energy level scheme in Figure 5. For fabricated xerogels, the intensities of recorded bands increased with decreasing in La^3+^:Dy^3+^ molar ratio from XG1 to XG5 sample, but for XG6 (with the highest content of Dy^3+^) the luminescence started to quench, suggesting the occurrence of the energy transfer (ET) process between neighboring Dy^3+^ ions in the host.

Generally, the relative intensities of the ^4^F_9/2_ → ^6^H_15/2_ (ΔJ = 3, forbidden transition) and the ^4^F_9/2_ → ^6^H_13/2_ emissions (ΔJ = 2, hypersensitive electric–dipole transition) are influenced by the symmetry in the nearest framework around Dy^3+^ ions [59]. Based on recorded spectra, *yellow-to-blue* (Y/B) ratios were calculated, and the obtained values were equaled to 2.83, 2.37, 2.27, 2.34, and 2.31 for XG2-6, respectively. For XG1 xerogel sample, the Y/B-ratio was not calculated due to the presence of a broad band in a blue light region with a maximum at λ = 434 nm (not shown in the figure), which coincides with the ^4^F_9/2_ → ^6^H_15/2_ emission of Dy^3+^ ions. The indicated background is associated with defects inside the amorphous sol-gel host, as was stated in the literature [60]. Indeed, it is attributed to photon recombinations from plentiful defects associated with dangling bonds inside the sol-gel skeleton, and its appearance is independent of the introduced rare-earth dopant, as was proven in our earlier works concentrated on Tb^3+^ and Eu^3+^ spectroscopies [46,47]. For the same reason (correlated with overlapping of this broad band with blue emission of Dy^3+^ ions), the Y/B-ratio for XG2 sample is higher than the values calculated for other XG3-XG6 samples characterized by greater intensities of emission lines from Dy^3+^ ions. Our experimental results for XG3-XG6 samples indicate that Y/B-ratio values are set at a nearly constant level, despite La^3+^:Dy^3+^ molar ratio and Dy^3+^ content, which suggests no significant changes in the local environment around optically active ions in samples before heat-treatment. In general, such high Y/B-ratio values obtained for precursor xerogels specify a relatively high covalent nature of bonds between Dy^3+^ and the host [13], and they are comparable with the values declared in the literature for selected amorphous systems depicted in Table 3 [12,59,61,62,63,64,65,66,67,68]. Indeed, similar Y/B-ratio values (above 2) have been reported for 35.7SiO_2_-25.5B_2_O_3_-17BaO-3.4K_2_O-3.4Al_2_O_3_-15BaCl_2_:0.1–1Dy_2_O_3_ [59], and 50B_2_O_3_-(25−x)CaO-15Al_2_O_3_-10CaF_2_-xDy_2_O_3_ (x = 0.5–5) [61] glassy systems. The data collected in Table 3 clearly indicate the strong correlation between Y/B-ratios and modifications in chemical compositions of glasses and amorphous sol-gel materials.

**Figure 6:**
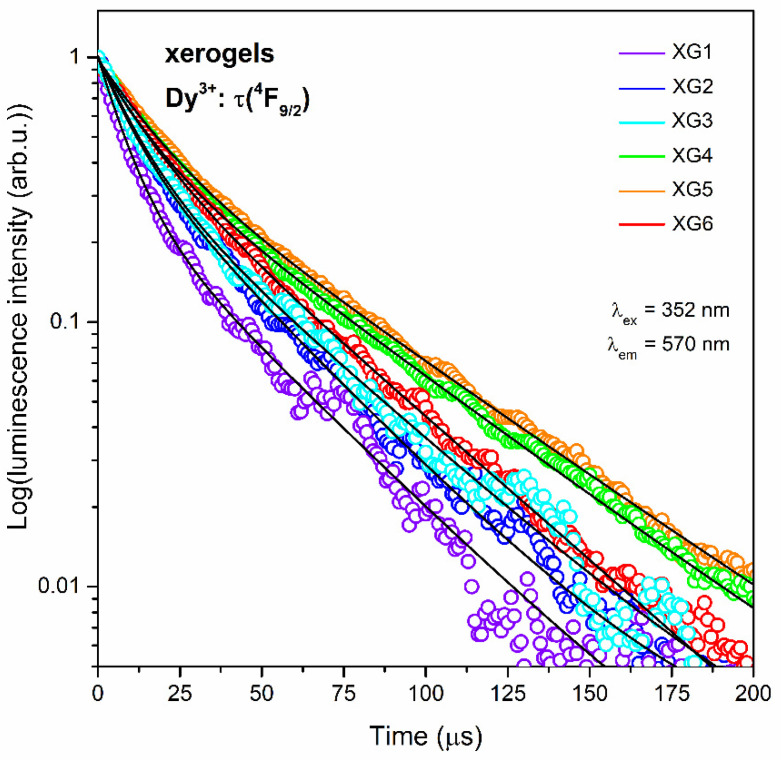
Luminescence decay curves recorded for the ^4^F_9/2_ state of Dy^3+^ ions in amorphous silicate xerogels (λ_ex_ = 352 nm, λ_em_ = 570 nm).

The further characterization of Dy^3+^-doped xerogels involved the luminescence decay analysis from the ^4^F_9/2_ excited state, and the resultant curves are presented in Figure 6 (λ_ex_ = 352 nm, λ_em_ = 570 nm). The registered luminescence decay curves followed the second-order exponential nature, and the average lifetimes were calculated using the following formula:

(3) $$\tau_{\mathrm{avg}}=\frac{A_{1}\tau_{1}^{2}+A_{2}\tau_{2}^{2}}{A_{1}\tau_{1}+A_{2}\tau_{2}}$$

where A_1_ and A_2_ are residual weighting factors and τ_1_ and τ_2_ are decay components. The resultant τ_n_(^4^F_9/2_):Dy^3+^ lifetimes with A_1_ and A_2_ parameters are depicted in Table 4.

The average decay times continuously elongate as the content of Dy^3+^ ions increased in the following order: 26.6 ± 0.7 (XG1), 28.9 ± 0.5 (XG2), 32.2 ± 0.8 (XG3), 40.6 ± 0.3 (XG4), and 42.7 ± 0.3 μs (XG5). However, for the sample with the highest content of Dy^3+^ ions (XG6), an evident shortening in the τ_avg_(^4^F_9/2_):Dy^3+^ value to 34.3 ± 0.1 μs was denoted, and it clearly corroborates with Dy^3+^-Dy^3+^ ET process. Generally, according to the numerous works in the literature, another factor that indicates the occurrence of the ET process among neighboring Dy^3+^ ions is the non-exponential behavior of the decays [12,14,64,67]. Based on this conception, we should assume that ET began to appear in the sample with the lowest content of Dy^3+^ (XG1), although its influence on the overall luminescence is negligible (indeed, we could observe the continuous elongation of the decays up to XG5 sample, simultaneously with growing intensities of the emission bands, as was presented in Figure 4). Therefore, for XG1-XG5 luminescence is proportional to the number of centers in an excited state. Further, for the XG6, the Dy^3+^-Dy^3+^ inter-ionic distances are the shortest in the series of fabricated xerogels, which makes the participation of ET enough to observe the shortening in the τ_avg_(^4^F_9/2_) lifetime value and quenching the emission. The τ(^4^F_9/2_):Dy^3+^ lifetimes reported in the current literature for other amorphous systems, i.e., calcium boroaluminate glasses (510–800 μs) [61] or zinc-alumino-borosilicate glasses (296.5–673.7 μs) [62] are significantly longer compared with the decay times obtained for xerogels in this work. However, we could assume that the observed tendency should be related to the limited content of OH groups in glassy hosts prepared by the melt-quenching technique (in comparison with xerogels), which play a crucial role in quenching of the luminescence originating from Dy^3+^ ions. Indeed, the τ_avg_(^4^F_9/2_):Dy^3+^ lifetimes for studied silicate xerogels are in the order of microseconds, and such relatively short luminescence lifetimes are strictly correlated with the presence of plentiful OH groups originated from silanol Si-OH moieties as well as residual organic solvents and water, inside a highly porous silicate network [44]. Since the ^4^F_9/2_ → ^4^F_1/2_ energy gap of Dy^3+^ ions equals only ΔE = ~7000 cm^−1^ [12], merely two high-energy phonons of OH groups (~3500 cm^−1^) are required to promote a non-radiative relaxation from the ^4^F_9/2_ excited state. As was also presented earlier by us for Eu^3+^ and Tb^3+^-doped samples [46], the non-radiative deactivation of the ^4^F_9/2_ level could also be partially caused by TFA ligands from RE^3+^ coordination sphere, containing carbonyl groups (~1665 cm^−1^; four groups to cover the energy gap) and C–F bonds (~1200 cm^−1^; six groups to cover the energy gap).

### 3.4. Luminescence Behavior of Dy^3+^-Doped Nano-Glass-Ceramics

**Figure 7:**
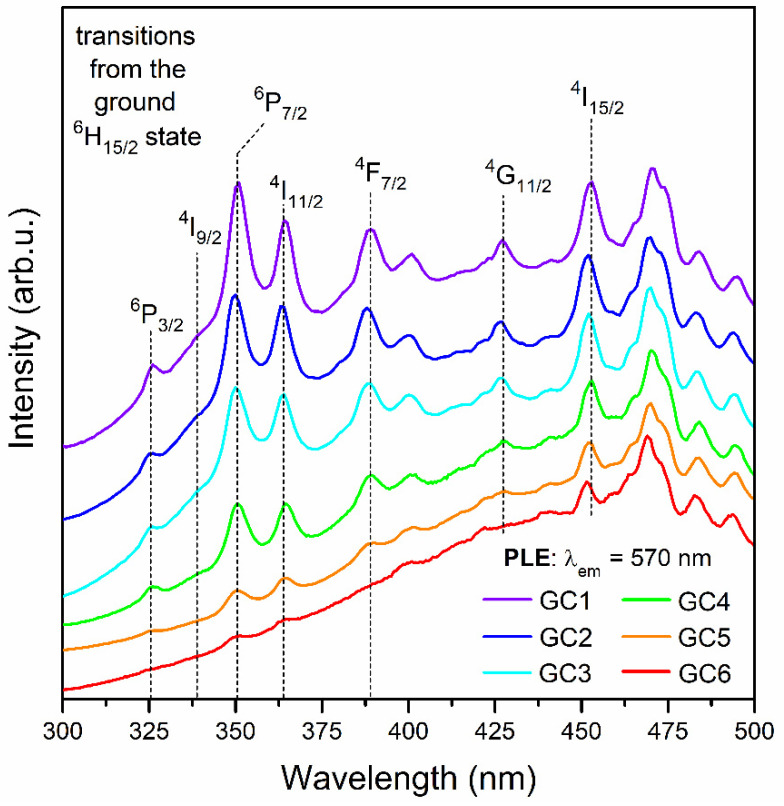
Photoluminescence excitation spectra (PLE) recorded for the series of fabricated SiO_2_-LaF_3_:Dy^3+^ nano-glass-ceramics by monitoring the yellow emission at λ_em_ = 570 nm.

Figure 7 shows the excitation spectra of SiO_2_-LaF_3_:Dy^3+^ nano-glass-ceramics, recorded by monitoring the characteristic yellow emission at λ_em_ = 570 nm. The spectra revealed the eight bands corresponding to the following electronic transitions: ^6^H_15/2_ → ^6^P_3/2_ (326 nm), ^6^H_15/2_ → ^4^I_9/2_ (339 nm), ^6^H_15/2_ → ^6^P_7/2_ (351 nm), ^6^H_15/2_ → ^4^I_11/2_ (364 nm), ^6^H_15/2_ → ^4^F_7/2_ (389 nm), ^6^H_15/2_ → ^6^G_11/2_ (427 nm), ^6^H_15/2_ → ^4^I_15/2_ (453 nm), and ^6^H_15/2_ → ^4^F_9/2_ (472 nm). Conversely to precursor xerogels, the excitation bands’ intensities gradually decrease with decreasing La^3+^:Dy^3+^ molar ratios in the subsequent GC1-GC6 samples. Hence, the concentration quenching phenomenon is observed from the nano-glass-ceramic with the lowest content of Dy^3+^ ions.

**Figure 8:**
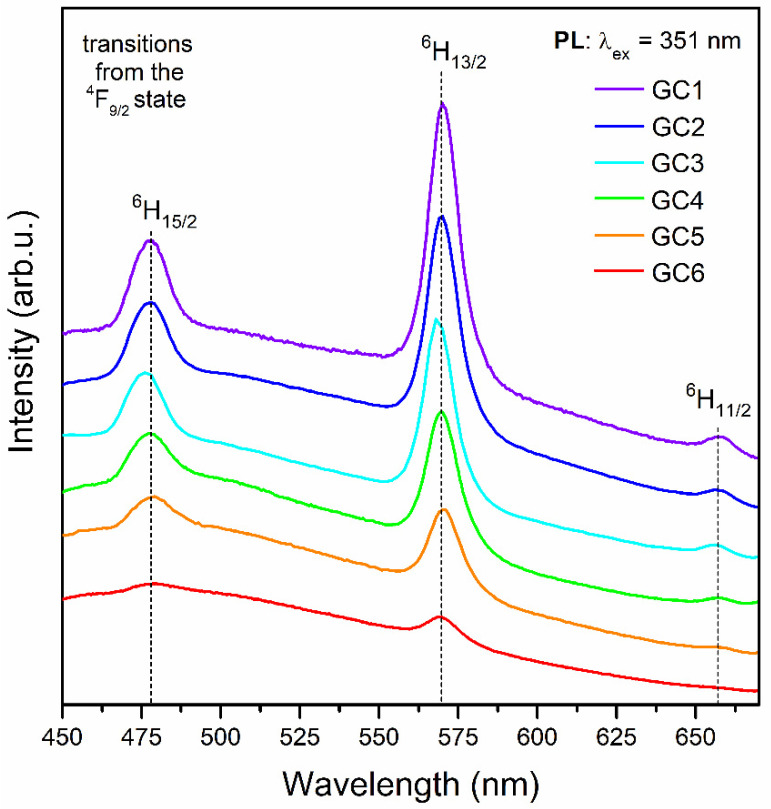
The photoluminescence emission (PL) spectra recorded for the series of prepared SiO_2_-LaF_3_:Dy^3+^ nano-glass-ceramics upon near-UV excitation at λ_em_ = 351 nm.

The emission spectra of Dy^3+^-doped nano-glass-ceramics, recorded upon excitation at λ_ex_ = 351 nm are presented in Figure 8. Similarly, as for xerogels, the characteristic emission bands of Dy^3+^ were identified at 478 nm (^4^F_9/2_ → ^6^H_15/2_), 570 nm (^4^F_9/2_ → ^6^H_13/2_), and 657 nm (^4^F_9/2_ → ^6^H_11/2_). A progressive decrease in the relative intensities of recorded bands for subsequent nano-glass-ceramics was observed. It could be stated that the concentration quenching has occurred even from the sample with the lowest content of Dy^3+^ ions (GC1), as was also observed in excitation spectra (Figure 7). Generally, the concentration quenching could be realized through the resonant energy transfer (RET) or possible non-radiative cross-relaxation channels CR1-CR3, as was presented in the energy level scheme in Figure 5. According to these channels, an excited Dy^3+^ ion (donor, D) makes a downward transition, whereas a coupled unexcited neighbor Dy^3+^ (acceptor, A) simultaneously makes an appropriate upward transition. The electronic transitions involved in each of the individual channel could be denoted as follows:

RET: ^4^F_9/2_ (D) + ^6^H_15/2_ (A) → ^6^H_15/2_ (D) + ^4^F_9/2_ (A),

CR1: ^4^F_9/2_ (D) + ^6^H_15/2_ (A) → (^6^H_9/2_ + ^6^F_11/2_) (D) + ^4^F_3/2_ (A),

CR2: ^4^F_9/2_ (D) + ^6^H_15/2_ (A) → ^6^F_5/2_ (D) + (^6^H_7/2_ + ^6^F_9/2_) (A),

CR3: ^4^F_9/2_ (D) + ^6^H_15/2_ (A) → ^6^F_3/2_ (D) + (^6^H_9/2_ + ^6^F_11/2_) (A).

As was discussed according to the gradual shift of XRD diffraction lines (Figure 2), we could expect that part of Dy^3+^ ions were entered into LaF_3_ nanocrystal lattice, which significantly promotes the shortening of the Dy^3+^-Dy^3+^ inter-ionic distances. The incorporation of Dy^3+^ ions inside fluoride nanophase could also be stated based on a decrease in Y/B-ratio values compared to those for precursor xerogels: 2.71 (GC1), 2.53 (GC2), 2.34 (GC3), 2.20 (GC4), 2.06 (GC5), and 1.94 (GC6). Indeed, the denoted alterations in Y/B-ratios indicate that the bonding between Dy^3+^ ions and the local environment is less covalent in prepared nano-glass-ceramics than in xerogels, but it should be noted that the verified decrease in calculated ratios is slight. Furthermore, compared with Y/B-ratios declared in the literature (Table 5 [25,64,69,70,71,72]), the values calculated for prepared SiO_2_-LaF_3_:Dy^3+^ materials remained relatively high. Thus, we could suppose that calculated Y/B-ratio values are correlated with the presence of a broad band attributed to the photon recombinations from structural defects inside the silicate sol-gel host (still visible even after controlled heat-treatment of xerogels; the maximum of this band was shifted from ~434 nm (before heat-treatment) to ~465 nm (after heat-treatment)), which directly overlaps with the characteristic emission lines of Dy^3+^ ions within the blue (^4^F_9/2_ → ^6^H_15/2_) and the green (^4^F_9/2_ → ^6^H_13/2_) light spectral scopes. On the other hand, a clear trend could be noticed according to gradually decreasing Y/B-ratios for the subsequent SiO_2_-LaF_3_:Dy^3+^ nano-glass-ceramics as the content of optically active Dy^3+^ ions grow. It suggests an increasing tendency to accumulate Dy^3+^ ions in the LaF_3_ phase, which is also confirmed by the continuous shift of the XRD diffraction lines.

**Figure 9:**
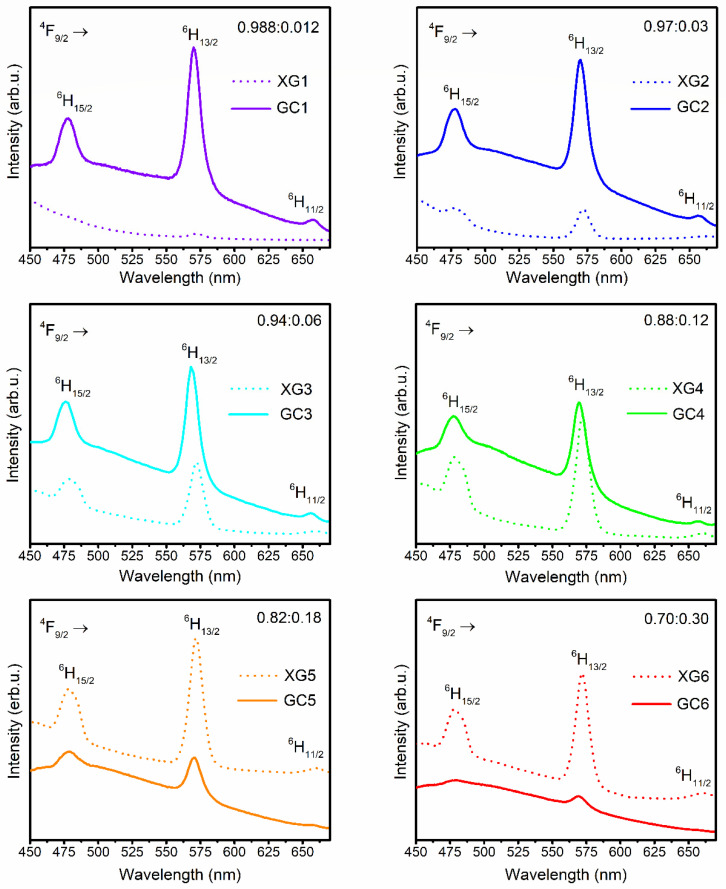
The comparison of emission spectra for xerogels and nano-glass-ceramic materials for individual La^3+^:Dy^3+^ molar ratios.

Additionally, the comparison of emission spectra recorded for xerogels and nano-glass-ceramics for individual La^3+^:Dy^3+^ molar ratios in samples’ compositions was presented in Figure 9. Based on this comparison, it can be stated that for La^3+^:Dy^3+^ molar ratios equal to 0.988:0.012, 0.97:0.03, and 0.94:0.06, the heat-treatment process enhances the intensity of the emission bands originated from Dy^3+^ ions. The most remarkable difference in the bands’ intensity can be observed when the content of Dy^3+^ is the lowest in the series of obtained samples (La^3+^:Dy^3+^ = 0.988:0.012). The correlation between luminescence intensities and La^3+^:Dy^3+^ molar ratio starts to change as the content of Dy^3+^ ions increases. For La^3+^:Dy^3+^ molar ratios equal to 0.88:0.12, 0.82:0.18, and 0.70:0.30, the emission intensities of luminescent bands of Dy^3+^ ions are greater for xerogels than for glass-ceramic materials. It is caused by the progressing concentration quenching, particularly for the highest content of Dy^3+^ ions, due to a significant shortening in the inter-ionic Dy^3+^-Dy^3+^ distances correlated with the incorporation of Dy^3+^ ions into the LaF_3_ fluoride phase.

**Figure 10:**
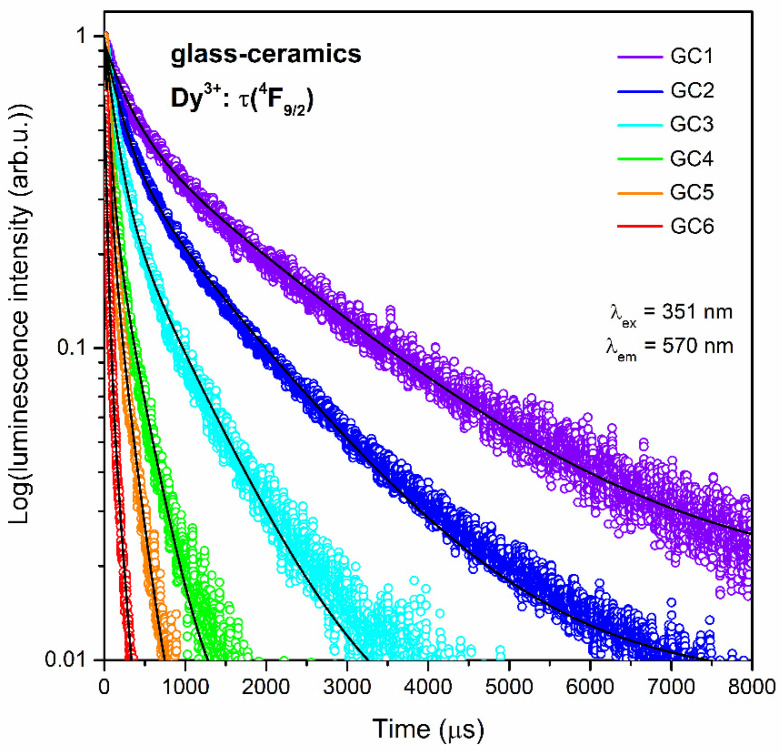
Luminescence decay curves recorded for the ^4^F_9/2_ state of Dy^3+^ ions for SiO_2_-LaF_3_ nano-glass-ceramic materials (λ_ex_ = 351 nm, λ_em_ = 570 nm).

The luminescence decay curves of the ^4^F_9/2_ state of Dy^3+^ for the series of prepared SiO_2_-LaF_3_ nano-glass-ceramics are illustrated in Figure 10. For all GCs, the decay curves follow the second-order exponential nature, which could, according to the distribution of Dy^3+^ ions, be either between a silicate xerogel host and fluoride nanocrystals with different decay rates, but could also indicate the ET process between neighboring Dy^3+^ ions in the host. The resultant τ_m_(^4^F_9/2_):Dy^3+^ lifetimes with A_1_ and A_2_ parameters are depicted in Table 6. Indeed, for the subsequent Dy^3+^-doped samples, the progressive shortening of the lifetimes was observed, and the average decay times equaled: 1731.5 ± 5.7 (GC1), 1124.1 ± 2.5 (GC2), 612.2 ± 3.0 (GC3), 232.0 ± 2.3 (GC4), 143.8 ± 1.5 (GC5), and 119.8 ± 0.4 μs (GC6). That denoted tendency to shortening of the decay times clearly indicates the continuous concentration quenching. It should also be noted that the ET is much more noticeable for nano-glass-ceramics than for xerogels (the shortening of the τ_avg_(^4^F_9/2_) was reported only for XG6 sample with La^3+^:Dy^3+^ molar ratio equals to 0.70:0.30), which is strictly associated with substantial decreasing in Dy^3+^-Dy^3+^ distances due to their partial entering into LaF_3_ nanocrystal lattice. Another noteworthy issue is related to the substantial elongation of the τ(^4^F_9/2_):Dy^3+^ lifetimes for nano-glass-ceramic materials in accordance with precursor xerogels, especially for samples with lower content of Dy^3+^, and it is associated with the low-phonon energy of LaF_3_ nanocrystal lattice (350 cm^−1^ [42]), which provides the low probability of depopulation of the excited states. Indeed, about 20 phonons of such fluoride phase would be needed to cover the energy gap between the ^4^F_9/2_ level and the ^6^F_1/2_ state of Dy^3+^ to quench the luminescence. Additionally, the remaining part of Dy^3+^ ions (which did not accumulate inside the fluoride lattice but are still located inside the amorphous sol-gel host) are surrounded by Q^3^ [SiO_4_] groups (1045 cm^−1^) with lower oscillation energy than OH moieties, which also reduces the probability of the ^4^F_9/2_ state depopulation. According to our previous research concentrated on the impact of structure on photoluminescence of RE^3+^ [46], it has been proven that the proposed thermal treatment conditions (350 °C/10 h) cannot trigger the complete elimination of OH groups from the sol-gel network; nevertheless, their amounts are significantly reduced compared to the xerogels. As a result, OH groups do not have a crucial impact on Dy^3+^ luminescence quenching. According to the literature data the τ(^4^F_9/2_):Dy^3+^ lifetimes for glass-ceramic materials with PbF_2_ [70], NaGd(WO_4_)_2_ [71], KNbO_3_ [73], or Ca_2_Ti_2_O_6_ [74] crystal phases do not exceed the value of 1 ms, while for studied SiO_2_-LaF_3_:Dy^3+^ nano-glass-ceramics (with lower contents of Dy^3+^ ions), longer lifetimes of about ~1.8 ms (GC1, La^3+^:Dy^3+^ = 0.988:0.012) and ~1.1 ms (GC2, La^3+^:Dy^3+^ = 0.97:0.03) were obtained. For higher contents of Dy^3+^ ions (when La^3+^:Dy^3+^ molar ratio equals 0.94:0.06, 0.88:0.12, and 0.70:0.30), the luminescence lifetimes are comparable with the values declared in the literature for those glass-ceramic systems [70,71,72,73,74].

Finally, it should be also pointed out that photoluminescence quantum yield (PLQY) is one of the essential spectroscopic parameters for RE^3+^-doped materials to judge their suitability for device fabrication, e.g., as visible light or infrared irradiation emitters. In the paper published by N. Maruyama et al. [75], the quantum yields for Dy^3+^-doped glass with 40BaO-20TiO_2_-40SiO_2_-0.5Dy_2_O_3_ and derivative nano-glass-ceramic were evaluated directly from measurements using an integrating sphere. As a result of the crystallization of precursor glasses, the intensities of emission bands according to the ^4^F_9/2_ → ^6^H_J_ (J = 15/2, 13/2, 11/2) transitions of Dy^3+^ ions significantly increased. As a result, the estimated quantum yield for Dy^3+^-doped nano-glass-ceramic is close to 15.2%, while for precursor glass it equaled 4.1%. Therefore, the quantum yield for nano-glass-ceramic is nearly 4-fold higher than for glass. Indeed, for Dy^3+^-doped sol-gel materials described in this work, the sum of the integrated intensities of individual blue (^4^F_9/2_ → ^6^H_15/2_), yellow (^4^F_9/2_ → ^6^H_13/2_), and red (^4^F_9/2_ → ^6^H_11/2_) emissions is at least 4.5-fold higher for SiO_2_-LaF_3_:Dy^3+^ nano-glass-ceramics compared with silicate xerogels before controlled heat-treatment. Nevertheless, it should be noted that this correlation is observed only for samples with low concentrations of Dy^3+^ ions in sol-gel hosts (with La^3+^:Dy^3+^ molar ratios equaled 0.988:0.012 and 0.97:0.03) when concentration quenching for glass-ceramics is inhibited. Thus, we believe that for those of fabricated nano-glass-ceramics, the quantum yield will be higher than for xerogels due to the preferable location of Dy^3+^ ions inside LaF_3_ fluoride nanocrystals and effective shortening of Dy^3+^-Dy^3+^ inter-ionic distances. These important aspects, according to the evaluation of luminescence quantum yields, will be examined in the future.

## 4. Conclusions

This paper presents the optical characterization of Dy^3+^-doped silicate xerogels and nano-glass-ceramics containing LaF_3_ phase, according to the structural modifications and variable La^3+^:Dy^3+^ molar ratios in the samples’ composition. The thermal degradation of La(TFA)_3_ and its transformation into the fluoride phase was verified by TG/DSC analysis, and XRD measurements confirmed the crystallization of LaF_3_ in the nanoscale. The luminescence characterization of prepared sol-gel samples involved the registration of excitation and emission spectra, along with the decay analysis from the ^4^F_9/2_ excited level of Dy^3+^. For amorphous xerogels, the concentration quenching occurs from the sample with the lowest proposed La^3+^:Dy^3+^ molar ratio (0.70:0.30, XG6), when the Dy^3+^-Dy^3+^ distances are the shortest in the series. The considerable differences in τ(^4^F_9/2_):Dy^3+^ lifetimes, the decrease in calculated Y/B-ratio, as well as the results from XRD analysis suggest the partial migration of Dy^3+^ from amorphous xerogel host into crystallized LaF_3_ nanophase during heat-treatment. Indeed, it was found that luminescence lifetimes are strongly dependent on Dy^3+^-Dy^3+^ inter-ionic distances determined by the content of optically active Dy^3+^ ions and the nature of prepared sol-gel materials (correlated with the vibrational energies in the immediate vicinity of optically active dopant). The embedding of Dy^3+^ inside LaF_3_ phase of prepared glass-ceramics resulted in continuous shortening of the inter-ionic distances, thus, the progressive quenching of the luminescence is observable even from the lowest content of Dy^3+^ (La^3+^:Dy^3+^ = 0.988:0.012, GC1). Simultaneously, the incorporation of Dy^3+^ into fluoride nanocrystals with low phonon energy resulted in substantial elongation of the τ(^4^F_9/2_) lifetimes compared with xerogels. The obtained results suggest that the fabricated Dy^3+^-doped materials could be predisposed for application as visible light emitters, like color screens or three-dimensional displays.

## Figures and Tables

**Table 1:** The parameters from TG, DTG, and DSC analysis for studied XG1-XG6 silicate xerogels doped with Dy^3+^ ions.

Sample	Thermal Degradation				
	1st Step		2nd Step		
	Temperature Range (°C)	Maximum of DTG Peak (°C)	Temperature Range (°C)	Maximum of DTG Peak (°C)	DSC Peak Position (°C)
XG1	55–223	129	223–383	307	307
XG2	56–222	134	222–382	303	302
XG3	50–224	135	224–382	307	307
XG4	57–226	157	226–378	291	298
XG5	51–217	132	217–386	307	307
XG6	45–201	111	201–375	307	309

**Table 2:** Crystal lattice parameters of LaF_3_ phase in prepared SiO_2_-LaF_3_:Dy^3+^ nano-glass-ceramics. The asterisk (*) refers to parameters of undoped LaF_3_ phase according to ICDD card no. 00-008-0461.

Sample	Lattice Parameter [Å]		Crystallite Size [nm]		Lattice Strain [%]
	LaF_3_ (*)	Sol-Gel Sample	Scherrer	Williamson–Hall	
GC1	a_0_ = 7.184 c_0_ = 7.351	a_0_ = 7.181(8) c_0_ = 7.359(4)	21.3 ± 0.5	10.6 ± 0.1	0.24 ± 0.01
GC2		a_0_ = 7.172(4) c_0_ = 7.351(9)	15.3 ± 0.3	8.2 ± 0.1	0.27 ± 0.01
GC3		a_0_ = 7.161(0) c_0_ = 7.343(3)	12.7 ± 0.1	9.0 ± 0.1	0.16 ± 0.01
GC4		a_0_ = 7.147(7) c_0_ = 7.321(9)	12.3 ± 0.1	9.0 ± 0.1	0.14 ± 0.01
GC5		a_0_ = 7.139(6) c_0_ = 7.311(5)	12.6 ± 0.1	9.8 ± 0.1	0.11 ± 0.01
GC6		a_0_ = 7.077(2) c_0_ = 7.242(9)	11.9 ± 0.1	9.0 ± 0.1	0.13 ± 0.01

**Table 3:** Y/B-ratios for different types of amorphous optical materials doped with Dy^3+^ ions.

Amorphous Material	Y/B-Ratio	Reference
35.7SiO_2_-25.5B_2_O_3_-17BaO-3.4K_2_O-3.4Al_2_O_3_-15BaCl_2_ (mol%):0.1–1wt% Dy_2_O_3_ ^2^	2.88–2.98	[59]
XG2-XG6 ^1^	2.83	[this work]
	2.37	[this work]
	2.27	[this work]
	2.34	[this work]
	2.31	[this work]
50B_2_O_3_-(25 − x)CaO-15Al_2_O_3_-10CaF_2_-xDy_2_O_3_ (x = 0.5–5) wt% ^2^	1.94–2.18	[61]
20SiO_2_-(40 − x)B_2_O_3_-10Al_2_O_3_-20NaF-10ZnO-xDy_2_O_3_ (x = 0.1–2.5) mol% ^2^	1.66–1.77	[62]
35B_2_O_3_-20SiO_2_-(15 − x)Al_2_O_3_-15ZnO-15Na_2_CO_3_-xDy_2_O_3_ (x = 0.1–2.5) mol% ^2^	1.61–1.75	[63]
45SiO_2_-20Al_2_O_3_-10CaO-24.9CaF_2_-0.1Dy_2_O_3_ mol% ^2^	1.52	[64]
73TeO_2_-4BaO-3Bi_2_O_3_-18SrF_2_-2Dy_2_O_3_ mol% ^2^	1.50	[65]
Ba_2_O_3_-PbO-Al_2_O_3_-WO_3_-Dy_2_O_3_ wt.% ^2^ (B_2_O_3_:PbO molar ratio changed from 2:1 to 1:8)	1.04–1.22	[66]
(20 − x)Na_2_O-5BaF_2_-5CaF_2_-60B_2_O_3_-10TeO_2_-xDy_2_O_3_ (x = 0.5–2.5) mol% ^2^	0.86–1.11	[12]
15ZnO-5PbO-(20 − x)Al_2_O_3_-60B_2_O_3_-xDy_2_O_3_ (x = 0.1–2.0) mol% ^2^	0.68–0.78	[67]
TEOS-based xerogels ^1^	0.51–0.76	[68]

^1^ materials prepared by the sol-gel method. ^2^ materials prepared by the conventional melt-quenching technique.

**Table 4:** Decay components (τ_n_), residual weighting factors (A_n_), and average decay times (τ_avg_) of the ^4^F_9/2_ state of Dy^3+^ in fabricated silicate xerogels.

Sample	Decay Components (μs)		Residual Weighting Factors (%)		Average Decay Time, τ_avg_ (μs)
	τ_1_	τ_2_	A_1_	A_2_	
XG1	8.0 ± 0.1	35.8 ± 0.8	68.76	31.24	26.6 ± 0.7
XG2	9.4 ± 0.2	34.3 ± 0.6	50.76	49.76	28.9 ± 0.5
XG3	10.8 ± 0.3	39.8 ± 0.9	56.79	43.21	32.2 ± 0.8
XG4	12.7 ± 0.1	47.8 ± 0.3	49.16	50.84	40.6 ± 0.3
XG5	15.0 ± 0.2	50.4 ± 0.3	48.28	51.72	42.7 ± 0.3
XG6	13.0 ± 0.1	40.3 ± 0.1	46.52	53.48	34.3 ± 0.1

**Table 5:** Y/B-ratios for glass-ceramics doped with Dy^3+^ ions.

Type of Crystal Phase	Y/B-Ratio	Reference
LaF_3_ ^1^ (350 °C)	2.74	[this work]
	2.53	
	2.34	
	2.20	
	2.06	
	1.94	
CaF_2_ ^2^ (650 °C, 700 °C)	1.58 1.68	[64]
β-NaGdF_4_ ^2^ (700 °C)	1.51	[69]
PbF_2_ ^2^ (380 °C/2 h)	1.18	[70]
PbF_2_ ^2^ (380 °C/5 h)	1.05	
PbF_2_ ^2^ (380 °C/10 h)	1.22	
NaGd(WO_4_)_2_ ^2^ (450 °C)	1.0–1.1	[71]
SrWO_4_ ^2^	0.787–0.881	[72]
Gd_2_(WO_4_)_3_ ^1,^*	0.23–1.37	[25]
La_2_(WO_4_)_3_ ^1,^*	0.26–1.21	[25]

^1^ materials prepared by sol-gel method. ^2^ materials prepared by conventional melt-quenching technique. * the crystal phase was not formed by in situ nucleation during controlled heat-treatment.

**Table 6:** Decay components (τ_n_), residual weighting factors (A_n_), and average decay times (τ_avg_) of the ^4^F_9/2_ level of Dy^3+^ in prepared nano-glass-ceramics containing LaF_3_ phase.

Sample	Decay Components (μs)		Residual Weighting Factors (%)		Average Decay Time, τ_avg_ (μs)
	τ_1_	τ_2_	A_1_	A_2_	
GC1	302.7 ± 2.1	1920.6 ± 6.8	45.64	54.36	1731.5 ± 5.7
GC2	223.3 ± 0.8	1317.8 ± 3.0	55.92	44.07	1124.1 ± 2.5
GC3	143.1 ± 0.6	782.5 ± 3.4	66.51	33.49	612.2 ± 3.0
GC4	64.2 ± 0.5	305.1 ± 2.6	67.44	32.56	232.0 ± 2.3
GC5	51.4 ± 0.4	197.6 ± 1.8	69.12	30.88	143.8 ± 1.5
GC6	42.9 ± 0.1	180.2 ± 0.5	76.76	23.24	119.8 ± 0.4

## Data Availability

The data presented in this study are available on request from the corresponding authors.

## References

[1] Archana L.S., Rajendran D.N. (2021). Luminescence of rare earth doped ZnS nanophosphors for the applications in optical displays. Mater. Today Proc..

[2] Frieiro J.L., Guillaume C., López-Vidrier J., Blázquez O., González-Torres S., Labbé C., Hernández S., Portier X., Garrido B. (2020). Toward RGB LEDs based on rare earth-doped ZnO. Nanotechnology.

[3] Liu Z., Ikesue A., Li J. (2021). Research progress and prospects of rare-earth doped sesquioxide laser ceramics. J. Eur. Ceram. Soc..

[4] Fois M., Cox T., Ratcliffe N., de Lacy Costello B. (2021). Rare earth doped metal oxide sensor for the multimodal detection of volatile organic compounds (VOCs). Sens. Actuators B.

[5] Mishra L., Sharma A., Vishwakarma A.K., Jha K., Jayasimhadri M., Ratnam B.V., Jang K., Rao A.S., Sinha R.K. (2016). White light emission and color tunability of dysprosium doped barium silicate glasses. J. Lumin..

[6] Jaidass N., Moorthi C.K., Babu A.M., Babu M.R. (2018). Luminescence properties of Dy^3+^ doped lithium zinc borosilicate glasses for photonic application. Heliyon.

[7] Rittisut W., Wantana N., Ruangtaweep Y., Mool-am-kha P., Padchasri J., Rujirawat S., Manyum P., Yimnirun R., Kidkhunthod P., Prasatkhetragarn A. (2021). Bright white light emission from (Gd^3+^/Dy^3+^) dual doped transparent lithium aluminum borate glasses for W- LED application. Opt. Mater..

[8] Wang L., Guo Z., Wang S., Zhang H., Lv H., Wang T., Su C. (2020). Luminescence properties of Dy^3+^ doped glass ceramics containing Na_3_Gd(PO_4_)_2_. J. Non-Cryst. Solids.

[9] Bu Y.Y., Cheng S.J., Wang X.F., Yan X.H. (2015). Optical thermometry based on luminescence behavior of Dy^3+^-doped transparent LaF_3_ glass ceramics. Appl. Phys. A.

[10] Komar J., Lisiecki R., Głowacki M., Berkowski M., Suszyńska M., Ryba-Romanowski W. (2020). Spectroscopic parameters of Dy^3+^ ions in La_3_Ga_0.5_Ta_0.5_O_14_ single crystal. J. Lumin..

[11] Lin X., Zhao L., Jiang B., Mao J., Chi F., Wang P., Xie C., Wei X., Chen Y., Yin M. (2019). Temperature-dependent luminescence of BaLaMgNbO_6_:Mn^4+^,Dy^3+^ phosphor for dual-mode optical thermometry. Opt. Mater..

[12] Murali Krishna V., Mahamuda S., Talewar R.A., Swapna K., Venkateswarlu M., Rao A.S. (2018). Dy^3+^ ions doped oxy-fluoro boro tellurite glasses for the prospective optoelectronic device applications. J. Alloys Compd..

[13] Pisarska J., Żur L., Pisarski W.A. (2011). Optical spectroscopy of Dy^3+^ ions in heavy metal lead-based glasses and glass-ceramics. J. Mol. Struct..

[14] Babu P., Jang K.H., Kim E.S., Shi L., Vijaya R., Lavín V., Jayasankar C.K., Seo H.J. (2010). Optical Properties and energy transfer of Dy^3+^-doped transparent oxyfluoride glasses and glass-ceramics. J. Non-Cryst. Solids.

[15] Mahamuda S., Syed F., Annapurna Devi C.B., Swapna K., Prasad M.V.V.K.S., Venkateswarlu M., Rao A.S. (2021). Spectral characterization of Dy^3+^ ions doped phosphate glasses for yellow laser applications. J. Non-Cryst. Solids.

[16] Maheshwari K., Rao A.S. (2022). Photoluminescence downshifting studies of thermally stable Dy^3+^ ions doped phosphate glasses for photonic device applications. Opt. Mater..

[17] Wu H., Liu X., Cao L., Zheng Y., Jin W., Li L. (2021). Effect of lattice distortion induced by Li^+^ doping on white light CaWO_4_:Dy^3+^ phosphors: Phase, photoluminescence and electronic structure. J. Lumin..

[18] Sheoran M., Sehrawat P., Kumari N., Khatkar S.P., Malik R.K. (2021). Cool white light emanation and photo physical features of combustion derived Dy^3+^ doped ternary yttrate oxide based nanophosphors for down converted WLEDs. Chem. Phys. Lett..

[19] Sehrawat P., Khatkar A., Boora P., Kumar M., Malik R.K., Khatkar S.P., Taxak V.B. (2020). Emanating cool white light emission from novel down-converted SrLaAlO_4_:Dy^3+^ nanophosphors for advanced optoelectronic applications. Ceram. Int..

[20] Eliseeva S.V., Salerno E.V., Lopez Bermudez B.A., Petoud S., Pecoraro V.L. (2020). Dy^3+^ White Light Emission Can Be Finely Controlled by Tuning the First Coordination Sphere of Ga^3+^/Dy^3+^ Metallacrown Complexes. J. Am. Chem. Soc..

[21] Anderson B.R., Gunawidjaja R., Eilers H. (2017). Dy^3+^-doped yttrium complex molecular crystals for two-color thermometry in heterogeneous materials. J. Lumin..

[22] Silva I.G.N., Kai J., Felinto M.C.F.C., Brito H.F. (2013). White emission phosphors based on Dy^3+^-doped into anhydrous rare-earth benzenetricarboxylate complexes. Opt. Mater..

[23] Grobelna B., Synak A., Głowaty D., Bojarski P., Szczodrowski K., Gryczyński I., Karczewski J. (2017). Novel inorganic xerogels doped with CaWO_4_:xDy: Synthesis, characterization and luminescence properties. Mater. Chem. Phys..

[24] Grobelna B., Synak A., Bojarski P. (2012). The luminescence properties of dysprosium ions in silica xerogel doped with Gd_1.6_Dy_0.4_(WO_4_)_3_. Opt. Appl..

[25] Grobelna B., Synak A., Bojarski P., Szczodrowski K., Kukliński B., Raut S., Gryczyński I. (2013). Synthesis and luminescence characteristics of Dy^3+^ ions in silica xerogels doped with Ln_2-x_Dy_x_(WO_4_)_3_. Opt. Mater..

[26] Santana-Alonso A., Yanes A.C., Méndez-Ramos J., del-Castillo J., Rodríguez V.D. (2011). Down-shifting by energy transfer in Dy^3+^-Tb^3+^ co-doped YF_3_-based sol-gel nano-glass-ceramics for photovoltaic applications. Opt. Mater..

[27] Velázquez J.J., Rodríguez V.D., Yanes A.C., del-Castillo J., Méndez-Ramos J. (2010). Increase in the Tb^3+^ green emission in SiO_2_-LaF_3_ nano-glass-ceramics by codoping with Dy^3+^ ions. J. Appl. Phys..

[28] Torres-Rodriguez J., Gutierrez-Cano V., Menelaou M., Kaštyl J., Cihlář J., Tkachenko S., González J.A., Kalmár J., Fábián I., Lázár I. (2019). Rare-Earth Zirconate Ln_2_Zr_2_O_7_ (Ln: La, Nd, Gd, and Dy) Powders, Xerogels, and Aerogels: Preparation, Structure, and Properties. Inorg. Chem..

[29] Cruz M.E., Durán A., Balda R., Fernández J., Mather G.C., Castro Y. (2020). A new sol–gel route towards Nd^3+^-doped SiO_2_-LaF_3_ glass-ceramics for photonic applications. Mater. Adv..

[30] Cheng S., Liu L., Yang Q., Li Y., Zeng S. (2019). In vivo optical bioimaging by using Nd-doped LaF_3_ luminescent nanorods in the second near-infrared window. J. Rare Earths.

[31] Wu H., Fei G.T., Gao J., Huang J., Zhang L.D. (2022). Single-Phase Organic−Inorganic Hybrid Nanoparticles for Warm-White Lighting. ACS Appl. Nano Mater..

[32] Chen Z., Wang W., Kang S., Cui W., Zhang H., Yu G., Wang T., Dong G., Jiang C., Zhou S. (2018). Tailorable Upconversion White Light Emission from Pr^3+^ Single-Doped Glass Ceramics via Simultaneous Dual-Lasers Excitation. Adv. Opt. Mater..

[33] Zhang H., Dong X., Jiang L., Yang Y., Cheng X., Zhao H. (2020). Comparative analysis of upconversion emission of LaF_3_:Er/Yb and LaOF:Er/Yb for temperature sensing. J. Mol. Struct..

[34] Peng Y., Zhong J., Li X., Chen J., Zhao J., Qiao X., Chen D. (2019). Controllable competitive nanocrystallization of La^3+^-based fluorides in aluminosilicate glasses and optical spectroscopy. J. Eur. Ceram. Soc..

[35] Ansari A.A., Parchur A.K., Labis J.P., Shar M.A. (2021). Physiochemical characterization of highly biocompatible, and colloidal LaF_3_:Yb/Er upconversion nanoparticles. Photochem. Photobiol. Sci..

[36] Nie L., Shen Y., Zhang X., Wang X., Liu B., Wang Y., Pan Y., Xie X., Huang L., Huang W. (2017). Selective synthesis of LaF_3_ and NaLaF_4_ nanocrystals via lanthanide ion doping. J. Mater. Chem. C.

[37] Chien H.-W., Huang C.-H., Yang C.-H., Wang T.-L. (2020). Synthesis, Optical Properties, and Sensing Applications of LaF_3_:Yb^3+^/Er^3+^/Ho^3+^/Tm^3+^ Upconversion Nanoparticles. Nanomaterials.

[38] Ansari A.A., Rai M., Rai S.B. (2017). Impact of LaF_3_ and silica shell formation on the crystal, optical and photo-luminescence properties of LaF_3_:Ce/Tb nanoparticles. Mater. Chem. Front..

[39] Mitroshenkov N.V., Matovnikov A.V., Kuznetsov S.V., Lazutkina M.V., Volchek A.A., Konoplin N.A., Kornev B.I., Novikov V.V. (2022). Low-temperature anomalies of thermodynamic properties of lanthanum trifluoride LaF_3_ and (SrF_2_)_0.5_(LaF_3_)_0.5_ multivalent solid solution. J. Alloys Compd..

[40] Hu M., Zhu Z., Wang Y., Li J., You Z., Tu C. (2018). Bulk Crystal Growth, First-Principles Calculations, and Mid-Infrared Spectral Properties of Dy^3+^ Doped and Dy^3+^/Nd^3+^ Codoped LaF_3_ Single Crystals. Cryst. Growth Des..

[41] Hong J., Zhang L., Hang Y. (2017). Enhanced 2.86 μm emission of Ho^3+^,Pr^3+^-codoped LaF_3_ single crystal. Opt. Mater. Express.

[42] Li S., Zhang L., Zhang P., Hong J., Xu M., Yan T., Ye N., Hang Y. (2017). Spectroscopic characterisations of Dy:LaF_3_ crystal. Infrared Phys. Technol..

[43] Cruz M.E., Castro Y., Durán A. (2022). Transparent oxyfluoride glass-ceramics obtained by different sol-gel routes. J. Sol-Gel Sci. Technol..

[44] Pawlik N., Szpikowska-Sroka B., Pietrasik E., Goryczka T., Pisarski W.A. (2018). Structural and luminescence properties of silica powders and transparent glass-ceramics containing LaF_3_:Eu^3+^ nanocrystals. J. Am. Ceram. Soc..

[45] Sun X., Zhang Y.W., Du Y.P., Yan Z.G., Si R., You L.P., Yan C.H. (2007). From Trifluoroacetate Complex precursors to Monodisperse Rare-Earth Fluoride and Oxyfluoride Nanocrystals with Diverse Shapes through Controlled Fluorination in Solution Phase. Chem. Eur. J..

[46] Pawlik N., Szpikowska-Sroka B., Goryczka T., Pisarska J., Pisarski W.A. (2021). Structural and Photoluminescence Investigations of Tb^3+^/Eu^3+^ Co-Doped Silicate Sol-Gel Glass-Ceramics Containing CaF_2_ Nanocrystals. Materials.

[47] Pawlik N., Szpikowska-Sroka B., Pietrasik E., Goryczka T., Pisarski W.A. (2019). Photoluminescence and energy transfer in transparent glass-ceramics based on GdF_3_:RE^3+^ (RE = Tb, Eu) nanocrystals. J. Rare Earths.

[48] Khan A.F., Yadav R., Singh S., Dutta V., Chawla S. (2010). Eu^3+^ doped silica xerogel luminescent layer having antireflection and spectrum modifying properties suitable for solar cell applications. Mater. Res. Bull..

[49] Pokhrel M., Gupta S.K., Perez A., Modak B., Modak P., Lewis L.A., Mao Y. (2021). Up- and Down-Convertible LaF_3_:Yb,Er Nanocrystals with a Broad Emission Window from 350 nm to 2.8 μm: Implications for Lighting Applications. Appl. Nano Mater..

[50] Lim D.J., Marks N.A., Rowles M.R. (2020). Universal Scherrer equation for graphene fragments. Carbon.

[51] Nath D., Singh F., Das R. (2020). X-ray diffraction analysis by Williamson-Hall, Halder-Wagner and size-strain plot methods of CdSe nanoparticles—A comparative study. Mater. Chem. Phys..

[52] D’Angelo P., Zitolo A., Migliorati V., Chillemi G., Duvail M., Vitorge P., Abadie S., Spezia R. (2011). Revised Ionic Radii of Lanthanoid(III) Ions in Aqueous Solution. Inorg. Chem..

[53] Sharma R.K., Mudring A.-V., Ghosh P. (2017). Recent trends in binary and ternary rare-earth fluoride nanophosphors: How structural and physical properties influence optical behavior. J. Lumin..

[54] Sharma R.K., Ghora M., Chouryal Y.N., Ganguly T., Acharjee D., Mondal D.J., Konar S., Nigam S., Ghosh P. (2022). Multifunctional Lanthanide-Doped Binary Fluorides and Graphene Oxide Nanocomposites Via a Task-Specific Ionic Liquids. ACS Omega.

[55] Kurian M., Kunjachan C. (2014). Investigation of size dependency on lattice strain of nanoceria particles synthesised by wet chemical methods. Int. Nano Lett..

[56] Prieur D., Bonani W., Popa K., Walter O., Kriegsman K.W., Engelhard M.H., Guo X., Eloirdi R., Gouder T., Beck A. (2020). Size Dependence of Lattice Parameter and Electronic Structure in CeO_2_ Nanoparticles. Inorg. Chem..

[57] Andrade A.B., Ferreira N.S., Valerio M.E.G. (2017). Particle size effects on structural and optical properties of BaF_2_ nanoparticles. RSC Adv..

[58] Leontyev I.N., Kuriganova A.B., Leontyev N.G., Hennet L., Rakhmatullin A., Smirnova N.V., Dmitriev V. (2014). Size dependence of the lattice parameters of carbon supported platinum nanoparticles: X-ray diffraction analysis and theoretical considerations. RSC Adv..

[59] Shasmal N., Karmakar B. (2019). White light-emitting Dy^3+^-doped transparent chloroborosilicate glass: Synthesis and optical properties. J. Asian Ceram. Soc..

[60] Kłonkowski A.M., Wiczk W., Ryl J., Szczodrowski K., Wileńska D. (2017). A white phosphor based on oxyfluoride nano-glass-ceramics co-doped with Eu^3+^ and Tb^3+^: Energy transfer study. J. Alloys Compd..

[61] Lodi T.A., Dantas N.F., Gonçalves T.S., de Camargo A.S.S., Pedrocki F., Steimacher A. (2019). Dy^3+^ doped calcium boroaluminate glasses and Blue Led for smart white light generation. J. Lumin..

[62] Monisha M., Mazumder N., Lakshminarayana G., Mandal S., Kamath S.D. (2021). Energy transfer and luminescence study of Dy^3+^ doped zinc-aluminoborosilicate glasses for white light emission. Ceram. Int..

[63] Bajaj R., Prasad A., Yeswanth A.V.S., Rohilla P., Kaur S., Rao A.S. (2022). Down-shifting photoluminescence studies of thermally stable Dy^3+^ ions doped borosilicate glasses for optoelectronic device applications. J. Mater. Sci. Mater. Electron..

[64] Babu P., Jang K.H., Rao C.S., Shi L., Jayasankar C.K., Lavín V., Seo H.J. (2011). White light generation in Dy^3+^-doped oxyfluoride glass and transparent glass-ceramics containing CaF_2_ nanocrystals. Opt. Express.

[65] Walas M., Lisowska M., Lewandowski T., Becerro A.I., Łapiński M., Synak A., Sadowski W., Kościelska B. (2019). From structure to luminescence investigation of oxyfluoride transparent glasses and glass-ceramics doped with Eu^3+^/Dy^3+^ ions. J. Alloys Compd..

[66] Górny A., Kuwik M., Pisarski W.A., Pisarska J. (2020). Lead Borate Glasses and Glass-Ceramics Singly Doped with Dy^3+^ for White LEDs. Materials.

[67] Deopa N., Saini S., Kaur S., Prasad A., Rao A.S. (2019). Spectroscopic investigations on Dy^3+^ ions doped zinc lead alumino borate glasses for photonic device application. J. Rare Earths.

[68] Thomas V., Jose G., Jose G., Biju P.R., Rajagopal S., Unnikrishnan N.V. (2005). Structural Evolution and Fluorescence Properties of Dy^3+^: Silica Matrix. J. Sol-Gel Sci. Technol..

[69] Sun X., Zhao S., Fei Y., Huang L., Xu S. (2014). Structure and optical properties of Dy^3+^/Tm^3+^ co-doped oxyfluoride glass ceramics containing β-NaGdF_4_ nanocrystals. Opt. Mater..

[70] Nageswara Rao C., Vasudeva Rao P., Kameswari R., Ramesh Raju R., Chandana G., Samatha K., Srinivas Prasad M.V.V.K., Venkateswarlu M., Naveen A., Dhar G.G. (2021). Luminescence investigations on Dy^3+^ doped CdO-PbF_2_ phosphate glass-ceramics. J. Mol. Struct..

[71] Lv H., Wang S., Su C., Zhang H., Guo Z., Wang L., Wang T., Wei Y. (2020). Preparation and luminescent properties of Dy^3+^-doped transparent glass-ceramics containing NaGd(WO_4_)_2_. J. Mater. Sci. Mater. Electron..

[72] Wei Y., Zhang H., Su C., Wang T., Wang S., Lv H., Zou X. (2020). Luminescence and preparation of Dy_2_O_3_ doped SrCO_3_-WO_3_-SiO_2_ glass ceramics. J. Lumin..

[73] Ramachari D., Rama Moorthy L., Jayasankar C.K. (2014). Energy transfer and photoluminescence properties of Dy^3+^/Tb^3+^ co-doped oxyfluorosilicate glass-ceramics for solid-state white lighting. Ceram. Int..

[74] Jia F., Xu S., Zhang G., Zhao T., Zou X., Zhang H. (2022). Effect of Mg^2+^/Sr^2+^ addition on luminescence properties of Dy^3+^ doped glass ceramics contining Ca_2_Ti_2_O_6_. Opt. Mater..

[75] Maruyama N., Honma T., Komatsu T. (2009). Enhanced quantum yield of yellow photoluminescence of Dy^3+^ ions in nonlinear optical Ba_2_TiSi_2_O_8_ nanocrystals formed in glass. J. Solid State. Chem..

